# Digital synthesis of histological stains using micro-structured and multiplexed virtual staining of label-free tissue

**DOI:** 10.1038/s41377-020-0315-y

**Published:** 2020-05-06

**Authors:** Yijie Zhang, Kevin de Haan, Yair Rivenson, Jingxi Li, Apostolos Delis, Aydogan Ozcan

**Affiliations:** 1 Electrical and Computer Engineering Department, University of California, Los Angeles, CA 90095 USA; 2 Bioengineering Department, University of California, Los Angeles, CA 90095 USA; 3 California NanoSystems Institute (CNSI), University of California, Los Angeles, CA 90095 USA; 4 Department of Computer Science, University of California, Los Angeles, CA 90095 USA; 5 Department of Surgery, David Geffen School of Medicine, University of California, Los Angeles, CA 90095 USA

**Keywords:** Wide-field fluorescence microscopy, Biophotonics

## Abstract

Histological staining is a vital step in diagnosing various diseases and has been used for more than a century to provide contrast in tissue sections, rendering the tissue constituents visible for microscopic analysis by medical experts. However, this process is time consuming, labour intensive, expensive and destructive to the specimen. Recently, the ability to virtually stain unlabelled tissue sections, entirely avoiding the histochemical staining step, has been demonstrated using tissue-stain-specific deep neural networks. Here, we present a new deep-learning-based framework that generates virtually stained images using label-free tissue images, in which different stains are merged following a micro-structure map defined by the user. This approach uses a single deep neural network that receives two different sources of information as its input: (1) autofluorescence images of the label-free tissue sample and (2) a “digital staining matrix”, which represents the desired microscopic map of the different stains to be virtually generated in the same tissue section. This digital staining matrix is also used to virtually blend existing stains, digitally synthesizing new histological stains. We trained and blindly tested this virtual-staining network using unlabelled kidney tissue sections to generate micro-structured combinations of haematoxylin and eosin (H&E), Jones’ silver stain, and Masson’s trichrome stain. Using a single network, this approach multiplexes the virtual staining of label-free tissue images with multiple types of stains and paves the way for synthesizing new digital histological stains that can be created in the same tissue cross section, which is currently not feasible with standard histochemical staining methods.

## Neural networks: Digital stains enhance pathologists’ arsenal

A machine learning approach uses combinations of multiple digital stains to highlight microscopic elements in a tissue sample, avoiding the delays, inconsistences and sometimes the need for multiple biopsies, all characteristic of traditional manual tissue staining techniques. The deep learning-based tissue staining framework was developed by Aydogan Ozcan, Yair Rivenson and colleagues at the University of California, Los Angeles. They used their approach to train neural networks to virtually stain kidney samples with one of three different types of stains or their combinations. Comparisons with manually stained tissue samples demonstrated that the virtual staining was highly accurate. The ability to use multiple digital stains and virtual stain blending on the same tissue sample using a single neural network could allow pathologists to get more relevant information from tissue and thus improve diagnoses.

## Introduction

Histological analysis is used to diagnose a wide variety of diseases. It is considered the gold standard for tissue-based diagnostics, with some well-established versions of common stains, such as haematoxylin and eosin (H&E), having been used for over a hundred years^1^. The histological staining process first requires the slicing of a fixed tissue specimen into sections of 2–10 μm, which are then fixed to microscope slides. Histological staining chemically introduces contrast into tissue sections, which can then be analysed and used to screen for diseases through bright-field microscopic imaging of the stained samples. However, histological staining can be a long and labour-intensive process, particularly in the case of special stains such as Jones’ silver stain and Masson’s trichrome stain. Therefore, the tissue staining process can increase both the time needed for diagnosis and the associated costs.

A wide variety of stains have been developed over the years to enable the visualization of different target tissue constituents. For example, haematoxylin stains cell nuclei, while Masson’s trichrome stain is used to view connective tissue^2^. These stains have also been chemically mixed to enable the visualization of different biomarkers. An example of this is when periodic acid-Schiff (PAS) and Alcian blue stains are used in conjunction to perform differential staining of glycoproteins^3^.

In recent years, various methods have been developed as substitutes for the histochemical staining of samples in an attempt to avoid (1) the specimen-destructive nature of the labelling process, allowing tissue preservation for more advanced analysis; (2) the lengthy and laborious labelling steps, saving time and cost; and (3) unnecessary additional biopsies from the same patient due to tissue depletion. Some of the earliest alternative contrast generation methods utilize various processes related to light-matter interaction, including nonlinear microscopy^4^, Raman scattering^5^, programmable supercontinuum pulses^6^ and reflectance confocal microscopy^7^. However, as pathologists (and, more recently, machine learning algorithms) are mainly trained to perform diagnoses using histologically stained specimens, images generated using alternative contrast mechanisms might require additional training to analyse. Recent efforts have also focused on the development of computational methods of creating bright-field microscopy images that closely resemble the stained versions of the same specimens. For example, digitally generated pseudo-stains have been demonstrated using analytical and statistical learning-based approaches that transform an input pixel (or pixel spectrum) into an RGB output pixel^5,8,9^. Some of these pixel-to-pixel transformation approaches have also used rapid staining methods to provide contrast in cell nuclei^4,9^.

Recently, emerging deep-learning methods have enabled the development of algorithms for learning accurate transformations between many different imaging modalities^10–14^. Notably, by utilizing the statistical correlations between the structures in images of unstained tissue slides and the structures in images of the same slides once stained, an unstained tissue sample can be virtually stained by a trained deep neural network without the need for any chemical processing. For example, using deep learning, autofluorescence images of unlabelled tissue samples have been virtually stained with various types of stains^15^. These virtual stains were validated through a blind study involving a team of board-certified pathologists, revealing that there is no statistically significant difference in quality between a virtually stained image and a standard histochemically stained version of the same sample imaged with a bright-field microscope in terms of either stain quality or diagnostic information. Various other techniques for performing virtual staining of unlabelled tissue images have also been demonstrated, for example, by using quantitative phase images^16^ or a combination of two-photon excitation and fluorescence lifetime imaging^17^. Researchers have also used deep learning to improve the accuracy of diagnosis using H&E images^18^; it has been shown that deep neural networks can be used to normalize stains, making them more consistent, which allows automated diagnostic analysis to be performed more easily^19^.

**Fig. 1:**
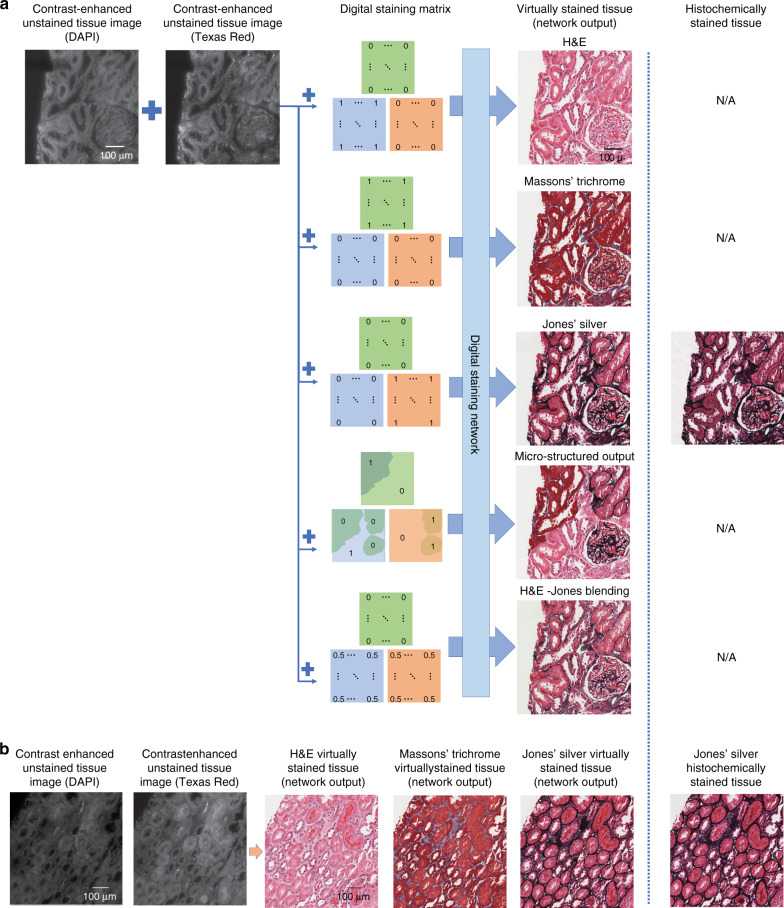
Demonstration of multiple stains being virtually generated using a class-conditional neural network and images in two autofluorescence channels (DAPI and Texas Red) of a label-free tissue sample. **a** Steps involved in virtually creating the various stains. By adding a class condition to the network using a digital staining matrix, a single network can be used to generate multiple stains or a blending of stains in the same tissue cross section on demand. **b** A second field of view demonstrating the three digital stains generated using a single trained network. Contrast-enhanced unstained tissue images are provided for visual guidance; unprocessed raw versions of these images were used as the input to the neural network. N/A (not available) refers to the fact that once a tissue section has been histochemically stained with one type of stain, we cannot subsequently stain it with other stains; therefore, the comparison includes N/A entries

In this paper, we demonstrate a novel machine-learning-based framework that allows users to virtually create micro-structured and multiplexed histological stains in the same tissue section using only a single artificial deep neural network. Using this technique, a trained deep neural network can (1) perform virtual staining in a defined region of interest following a micro-structure map defined by the user and (2) achieve the blending of multiple virtual stains and the synthesis of new digital stains. This framework uses the stain type as the input class for a conditional generative adversarial network (GAN) to transform the input images, consisting of pairs of unlabelled autofluorescence images of the same tissue sample, into a virtually stained image of the same label-free sample. To do so, we introduce a “digital staining matrix”, which is used as part of the input to the deep network, to spatially encode the stain type, i.e., each pixel can be virtually stained using a different stain type or a different set of histological stains (see Fig. 1).

To demonstrate the utility of this technique, we have trained a single neural network to virtually stain unlabelled autofluorescence images of kidney needle core biopsy tissue sections with H&E, Jones’ silver stain, and Masson’s trichrome stain following a user-defined micro-structure map, as illustrated in Fig. 1. Synthesizing different histological stains and their combinations following a user-defined micro-structure in the same tissue section is currently not feasible with a standard histological staining process, in which different stains are histochemically processed in different tissue sections, leading to tissue depletion. Our approach entirely eliminates the need for this, preserving tissue for further analysis while also paving the way for the on-demand synthesis of new digital histological stains in the same tissue section.

## Results

**Fig. 2:**
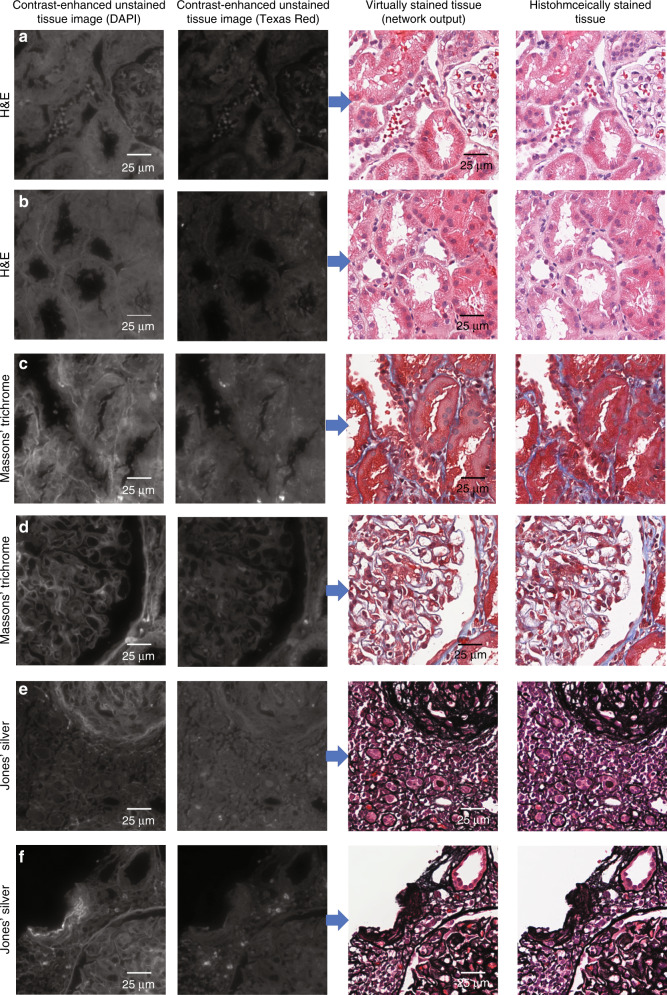
Examples of various fields of view that have been virtually stained using the presented multistain network. Co-registered histochemically stained fields of view of the same samples are also shown to the right, and the unstained autofluorescence images are shown to the left to permit direct comparison. **a**, **b** Tissue stained with H&E, **c**, **d** tissue stained with Masson’s trichrome, and **e**, **f** tissue stained with Jones’ silver. Contrast-enhanced unstained tissue images are provided for visual guidance; unprocessed raw versions of these images were used as the input to the neural network

As summarized in Fig. 1, we demonstrate a method that can be used to perform virtual staining of unlabelled tissue sections using two channels of tissue autofluorescence along with a digital staining matrix, which are used as inputs to a trained deep neural network. We choose to demonstrate the framework using kidney tissue and three different stains, namely, H&E, Masson’s trichrome, and Jones’ silver stain, as these stains are jointly used for practical renal disease diagnostics. Visualizations of comparisons between histochemically and virtually stained tissue sections can be seen in Figs. 1 and 2.

We validated the accuracy of the network inference outcomes using quantitative metrics. We calculated these quantitative metrics to confirm that the images generated by the multistain network are highly accurate and that they are equivalent to the images generated using a previously validated single-stain neural network^15^. The first quantitative metric used is the structural similarity index^20^ (SSIM), which is defined as:

1 $${\mathrm{SSIM}}\left( {a,b} \right) = \frac{(2\mu _a\mu _b + C_1)(2\sigma _{a,b} + C_2)}{({\mu _a}\,^2 + {\mu _b}\,^2 + C_1)({\sigma _a}\,^2 + {\sigma _b}\,^2 + C_2)}$$

where *μ*_*a*_ and *μ*_*b*_ are the averages of *a* and *b*, the two images being compared; *σ*_*a*_ and *σ*_*b*_ are the standard deviations of *a* and *b*; *σ*_*ab*_ is the cross-covariance of *a* and *b*; and *C*_*1*_ and *C*_*2*_ are stabilization constants that are used to avoid division by zero.

Table 1 reports the average SSIM values across four unique blindly tested kidney tissue blocks, each from a different patient. Each of these blocks, in turn, was divided into 16–60 patches (1224 × 1224 pixels, or 0.16 mm^2^ per patch), each comprising an unlabelled autofluorescence image pair and its co-registered histochemically stained counterpart (see the ‘Materials and methods' section). Because this comparison relies on histochemical staining of the same tissue section, a different section from each tissue block was used for each of the three different stain types. The variation in the number of patches is partially due to the variations in the size of the tissue blocks among patients; furthermore, images that could not be successfully co-registered due to, for example, histochemical-staining-induced tissue distortions were excluded from the SSIM calculations. Three different SSIM values were calculated for each stain type to prove that this new virtual staining technique is successful: (1) the SSIM between the output image of the conditional multistain network and the corresponding image of the histochemically stained tissue, (2) the SSIM between the output of a previously validated^15^ single-stain network architecture (see the ‘Materials and methods' section) and the corresponding image of the histochemically stained tissue, and (3) the SSIM between the outputs of the multistain network and the single-stain network for each of the three stains. As shown in Table 1, a high structural similarity is found for all three cases. Furthermore, the SSIM values calculated for cases (1) and (2) are found to be very similar, indicating that the images generated by the multistain network achieve the same virtual staining performance as was previously reported and validated using the single-stain network^15^. The particularly high structural similarity between the two different virtual staining techniques, i.e., case (3), is also important because the corresponding images are perfectly co-registered since they were generated from the same raw fluorescence images. Together, these results suggest that the presented multistain network generates highly accurate virtually stained images. This demonstrates that the images generated by the multistain network are similar to the corresponding bright-field images of histochemically stained tissue and are of the same quality as those generated by the single-stain network.

**Table 1:** Comparison of SSIM values among the outputs of the different networks and the corresponding histochemically stained tissue images

Stain type	(1) Multistain network output vs. histochemically stained tissue		(2) Single-stain network output vs. histochemically stained tissue		(3) Multistain network output vs. single-stain network output		Total number of image patches compared
	Average	Standard deviation	Average	Standard deviation	Average	Standard deviation	
H&E	0.898	0.021	0.905	0.022	0.967	0.006	198
Masson’s trichrome	0.850	0.011	0.855	0.023	0.942	0.010	207
Jones’ silver	0.803	0.007	0.803	0.010	0.917	0.007	118

The averages and standard deviations were calculated across four measured tissue sections

These different sets of comparisons between SSIM values are required because the SSIM values between any virtually stained and histochemically stained images depend on a number of factors, some of which are external to the performance of the trained neural network. Perfect co-registration is not feasible, particularly since physical changes are made to the tissue during the actual staining process^15^, somewhat lowering the structural similarity values regardless of the success of the virtual staining network. Furthermore, one of the major benefits of deep-learning-based virtual staining is stain normalization, as the network output will not exhibit the staining variability of the standard histochemical staining process as performed by histotechnologists^15^. While this is certainly a desired feature and will help to improve the consistency of diagnoses, it also lowers the SSIM values due to the histotechnologist-to-histotechnologist variations that are encountered in our ground-truth images.

As another quantitative metric, we next compared the average percentage differences in the brightness and chroma components (using the YCbCr colour space) for the three cases reported in Table 1, i.e., (1) multistain network output vs. histochemically stained tissue, (2) single-stain network output vs. histochemically stained tissue, and (3) multistain network output vs. single-stain network output. As summarized in Table 2, similar to the case of the SSIM values, the colour differences for cases (1) and (2) are very similar. Case (3) shows particularly small differences, indicating that the two networks (multistain and single-stain) behave very similarly. The change in the brightness (Y) of the multistain network output with respect to the histochemically stained tissue images is relatively low, ranging from 3.84 to 8.57% depending on the stain. The colour distances (Cb and Cr differences) are even smaller (ranging from 0.51 to 2.60% depending on the stain type; see Table 2), indicating that the multistain network accurately generates the correct colours that represent each stain. Together, these results further demonstrate that the multistain network is capable of accurate virtual staining of unlabelled autofluorescence images of tissue samples and that the output of the multistain network matches the accuracy of the previously validated tissue- and stain-specific neural network^15^.

**Table 2:** Comparison of brightness and chroma differences (using the YCbCr colour space) between (1) multistain network output and histochemically stained tissue, (2) single-stain network output and histochemically stained tissue, and (3) multistain network output and single-stain network output

Stain type	Comparison	Y difference (%)		Cb difference (%)		Cr difference (%)		Total number of image patches compared
		Average	Standard deviation	Average	Standard deviation	Average	Standard deviation	
H&E	(1) Multistain network vs. histochemically stained tissue	6.62	3.32	0.51	0.18	1.69	1.18	198
	(2) Single-stain network vs. histochemically stained tissue	7.78	3.48	0.87	0.21	2.04	1.41	
	(3) Multistain network vs. single-stain network	1.48	0.12	0.22	0.03	0.72	0.20	
Masson’s trichrome	(1) Multistain network vs. histochemically stained tissue	3.85	1.50	1.34	0.87	2.60	1.16	207
	(2) Single-stain network vs. histochemically stained tissue	5.31	1.32	2.09	1.51	3.00	1.44	
	(3) Multistain network vs. single-stain network	1.96	1.70	0.43	0.19	1.35	0.53	
Jones’ silver	(1) Multistain network vs. histochemically stained tissue	8.56	2.01	0.82	0.12	2.45	0.69	118
	(2) Single-stain network vs. histochemically stained tissue	9.07	1.93	1.33	0.21	3.15	0.86	
	(3) Multistain network vs. single-stain network	4.32	1.01	0.34	0.11	1.15	0.24	

The averages and standard deviations were calculated across four measured tissue sections

**Fig. 3:**
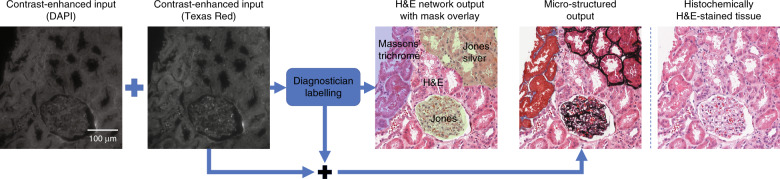
Example of multistain micro-structuring. Either a diagnostician or an algorithm can label sub-regions of the unstained tissue, creating on demand a digital staining matrix that defines the microscopic map of multiple stains to be virtually generated in the same tissue section. These labels are used by a single trained network to stain different areas of the tissue with the desired stains. A co-registered image of the histochemically H&E-stained tissue (same sample) is shown for comparison. Producing a histochemically stained image with the same or a similar microscopic map, with multiple stains in the same tissue section, is not possible with current chemical staining technology

One of the major advantages of using a class-conditional neural network is that it can perform micro-structured virtual staining of tissue sections. Using the presented method, virtual staining of specific areas or structures within tissue sections can be performed by staining different areas of the tissue in accordance with a given micro-structure map. The digital staining matrix, which defines the micro-structure map used to virtually apply different stains for each specified area, can be generated either manually or through the use of a computer algorithm to select structures based on certain diagnostic criteria. An example of virtual stain micro-structuring in accordance with a manually drawn micro-structure map is shown in Fig. 3. In this example, the marked areas are virtually stained with Masson’s trichrome and Jones’s silver stains, while the remaining areas not selected are stained with H&E. A co-registered image of the same field of view (FOV) after histochemical H&E staining is also shown for comparison.

**Fig. 4:**
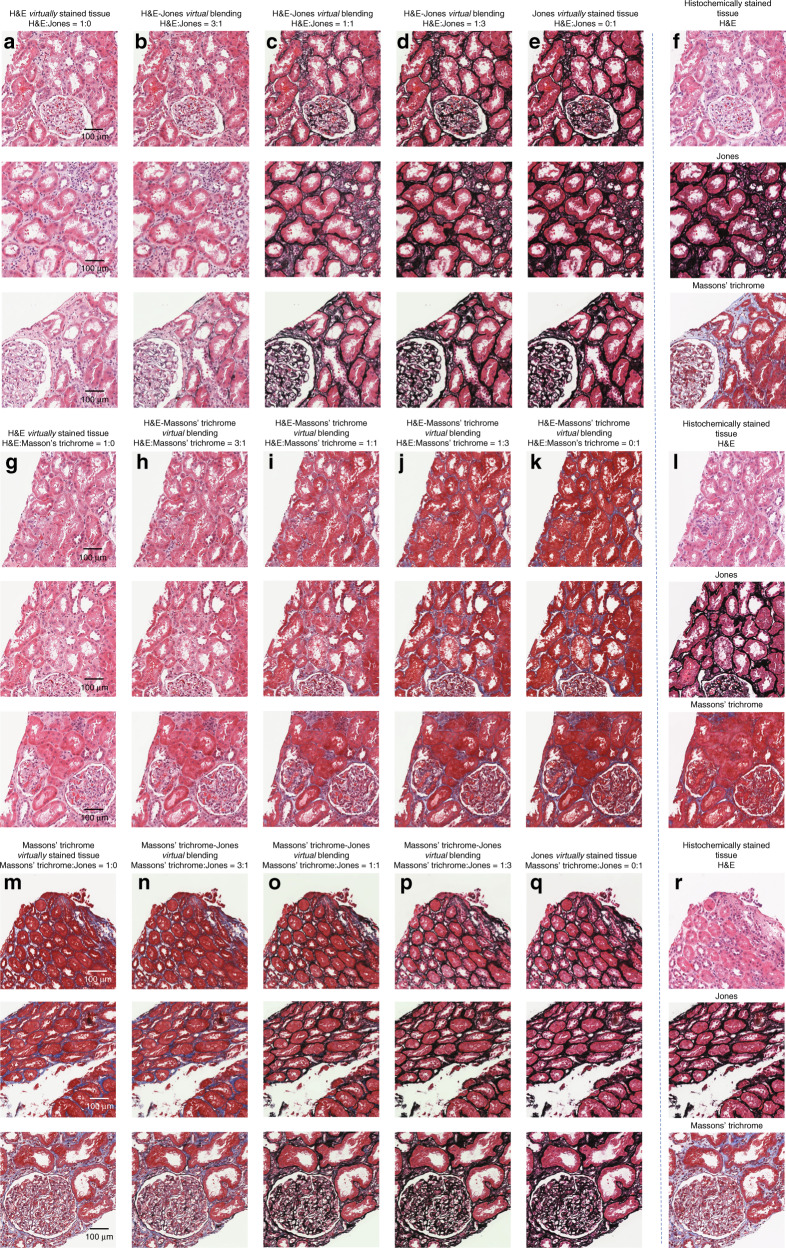
Examples of stain blending. **a**–**e** Kidney tissue that has been virtually stained with varying class-condition ratios of H&E to Jones’ silver stain. **g**–**k** Kidney tissue that has been virtually stained with varying class-condition ratios of H&E to Masson’s trichrome stain. **m**–**q** Kidney tissue that has been virtually stained with varying class-condition ratios of Masson’s trichrome stain to Jones’ silver stain. **f**, **l**, **r** Co-registered images of the histochemically stained tissues (same samples) for comparison (top: H&E; middle: Jones’ silver stain; bottom: Masson’s trichrome stain)

Stain blending can also be used to digitally synthesize new types of stains. Rather than using the digital staining matrix to generate individual stains, a mixture of multiple stains can be chosen. Such a stain mixture is generated by designing the digital staining matrix to mix two or more stains in the desired tissue areas, simultaneously and at controllable ratios (see Fig. 4). In other words, the newly generated stain can be tuned on demand by simply changing the ratio between the different values in the digital staining matrix, thus making the different stain combinations more or less pronounced. Figure 4 demonstrates several such stain combinations for different pairs of stains. By using these blended stains, aspects of the different stains can be made visible at the same time, which may allow pathologists to more easily view different tissue structures and perform diagnosis. For example, Fig. 4 a–e demonstrates blending between H&E and Jones’ silver stain; H&E enables easy differentiation of cell nuclei and cytoplasms^21^, while Jones’ silver stain provides contrast to basement membranes^22^. By blending these two stains, their associated characteristics can be visualized simultaneously.

While the digital stains generated here are completely unique to virtual staining, various histochemical stains have also been mixed together to generate new stain combinations^3^. However, new chemically developed stain combinations can take a large amount of time and resources to reach maturity. In contrast, the stain blending combinations presented here can be developed on demand by simply changing the values of the digital staining matrix until the desired stain is achieved. The virtual stain blending presented here also has a different effect on the tissue than standard histochemical stains, introducing a new mode of micro-structured visualization for pathologists.

## Discussion

In this paper, we have demonstrated that autofluorescence images of a label-free tissue sample can be used to perform micro-structured and multiplexed virtual staining using a deep neural network. By adding a digital staining matrix to the input of the neural network, we can generate multiple virtual stains upon the same tissue section using a single network. The success of this approach has been validated using kidney tissue sections and three different stains—H&E, Masson’s trichrome stain and Jones’ silver stain—and allows a pathologist to view the same areas of a sample with all three stains, perfectly matched in the same tissue cross section. The digital staining matrix also allows us to perform micro-structured virtual staining of a label-free sample, in which the sub-area for each stain can be defined either manually or using a separate algorithm. This approach can further be used to perform stain blending by using a digital combination of the stains that the multistain neural network has been trained to apply.

The ability to apply multiple stains to a single tissue section using a single neural network, alongside the newly added capabilities of stain blending, synthesis, and micro-structured virtual staining, has the potential to improve the accuracy and consistency of tissue-based diagnoses. These new techniques might allow pathologists to obtain more relevant information from tissue than is otherwise possible. By applying stains to specific areas, each tissue constituent can be stained with the most relevant stain. By blending stains, the network is able to simultaneously display information conveyed by each of the separate stains, providing additional channels of information to the pathologists making diagnoses.

These virtual staining techniques also open up opportunities to augment the diagnostic workflow currently used by pathologists and/or machine-learning-based diagnostic algorithms. Virtual staining normalizes the stain quality, improving its consistency and removing variations (caused by, for example, the manual histochemical staining performed by trained professionals) that have not been learned by the neural network^15^. Furthermore, micro-structured staining and stain blending can ensure that the diagnostic platform has access to the most relevant information possible, reducing the amount of unnecessary data viewed/processed by either a pathologist or an algorithm. Consequently, we believe that the push-pull relationship between the presented virtual staining framework and diagnosticians (human or AI-based) will lead to new uses of the capabilities of this unique framework in pathology and clinical diagnosis, all of which must be clinically validated through rigorous testing and blinded large-scale studies.

## Materials and methods

### Data acquisition

Unstained formalin-fixed and paraffin-embedded (FFPE) kidney tissues were sectioned into thin, 2-μm slices and fixed on standard glass microscope slides. The training and validation dataset for each stain was made up of images obtained from 12-thin tissue sections acquired from unique patients. The test dataset was made up of four tissue sections from additional unique patients. Adjacent tissue sections from each of these patients were used for each of the three stains. Ethical approval for the use of these tissue sections was obtained under UCLA IRB number 18–001029. Using a conventional widefield fluorescence microscope (IX83, Olympus) equipped with a 20×/0.75 NA objective lens (Olympus UPLSAPO) and two separate filter cubes, DAPI (OSFI3-DAPI-5060C, EX 377/50 nm EM 447/60 nm, Semrock) and Texas Red (OSFI3-TXRED-4040C, EX 562/40 nm EM 624/40 nm, Semrock), autofluorescence imaging of these unlabelled tissue sections was performed. The tissue sections were neither deparaffinized nor cover-slipped before being imaged via fluorescence microscopy. The exposure time for the DAPI channel was 50 ms, and that for the Texas Red channel was 300 ms. Once the autofluorescence images had been obtained, the slides were histochemically stained using standard H&E, Jones’ silver or Masson’s trichrome stain and were then cover-slipped. The staining of the slides was performed by the UCLA Translational Pathology Core Laboratory (TPCL). The histochemically stained slides were then imaged using a scanning microscope (Aperio AT, Leica Biosystems, 20×/0.75NA objective with a 2× adapter) to create the target labels used to train, validate and test our neural network models.

We used the two unlabelled autofluorescence images of the same tissue sample in conjunction with a digital staining matrix to select the stain or set of stains to be generated as the input to a neural network. This input was transformed by a class-conditional generative adversarial network into an equivalent image of a stained tissue section with the same field of view.

### Image pre-processing and co-registration

Because the purpose of the deep neural network was to learn the transformation from the unlabelled autofluorescence images of a tissue specimen to an image of a stained specimen, it was crucial that the FOVs were accurately aligned. Furthermore, since more than one autofluorescence channel was used as the network input, it was necessary to align the different filter channels. To use three different stains (H&E, Masson’s trichrome and Jones’s silver), we implemented image pre-processing and alignment for each pair of input and target images from the three staining datasets individually.

**Fig. 5:**
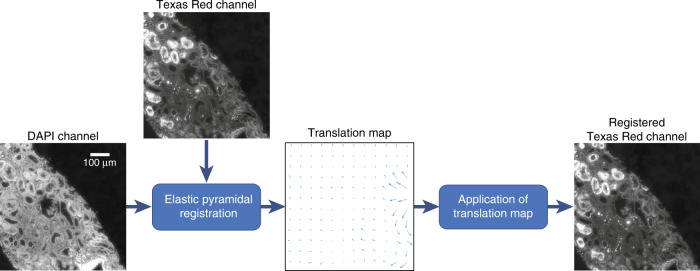
Example of co-registration between the DAPI and Texas Red image channels. The translation map was calculated using an elastic pyramidal registration algorithm and then applied to the Texas Red image channel. This process ensured that the two image channels were accurately co-registered with respect to each other

The registration steps for matching the autofluorescence and bright-field images followed the process reported by Rivenson et al.^15^. One major addition is that when multiple autofluorescence channels (e.g., DAPI and Texas Red) are used as the network input, they must be aligned even if the images in both channels are captured using the same microscope; the corresponding FOVs from the two channels are not precisely aligned at the subpixel level, particularly at the edges of the FOVs. Therefore, we applied an elastic pyramidal registration algorithm to accurately align the multiple autofluorescence channels. This elastic registration algorithm matches the local features of two image channels by hierarchically breaking the image into increasingly smaller blocks and then matching the corresponding blocks^10^. The elastic registration algorithm begins by dividing the image into a grid of 5 × 5 blocks and calculating block-wise cross-correlations. The distance between the location with the peak correlation and the centre of the block is used to calculate the shift, as the area with the peak correlation is the point with maximum similarity between the two images. By using a weighted average of the translation vector for each block, a 2048 × 2048-pixel translation map was generated. This translation map was then applied to the Texas Red image to account for the differences between it and the DAPI image. To achieve accurate co-registration, the image was iteratively broken into increasingly smaller blocks until a block size of 100 × 100 pixels was reached. The final calculated transformation map was then applied to the Texas Red images to ensure that they were aligned with the corresponding images in the DAPI channel. An example of the use of this elastic transformation map can be seen in Fig. 5. Finally, we stitched the aligned images from both channels to obtain whole-slide images of the samples that contained both the DAPI and Texas Red channels.

Co-registration between the fluorescence and bright-field images began with global registration and proceeded with progressive alignment at smaller scales until subpixel-level co-registration was achieved. This first step of this process was to find a rough match by extracting the area of the bright-field image with the highest cross-correlation with a contrast-reversed version of the DAPI image. These images were then further aligned using MATLAB’s multimodal image registration feature^23^. Following this registration process, the neural network was trained using patches from these coarsely matched images. Using this network, the autofluorescence images were then virtually stained. However, because only coarse co-registration had been achieved at this point, the accuracy of the virtual staining results could not be satisfactory. Therefore, elastic pyramidal co-registration was then applied to match the histochemically stained images with the initial virtually stained images, resulting in a matched image pair.

Before feeding the aligned pairs into the neural network, we implemented normalization on the whole-slide images generated from the DAPI and Texas Red images. This whole-slide normalization was performed by subtracting the mean value of the entire tissue sample and dividing by the standard deviation of the pixel values (note that background regions were excluded when calculating the mean and standard deviation).

### Deep neural network architecture, training and validation

In this study, we used a class-conditional GAN architecture to learn the transformation from the label-free unstained autofluorescence input images to the corresponding bright-field image using three different stains (H&E, Masson’s trichrome and Jones’ silver). Following the co-registration of the autofluorescence images and the bright-field images, the accurately aligned FOVs were randomly partitioned into overlapping patches of 256 × 256 pixels and further augmented through rotation and flipping. The patches were then used to train the GAN. During the training process, this class-conditional GAN used a set of one-hot-encoded matrices, together referred to as the digital staining matrix, which was concatenated with the network’s 256 × 256 input image/image stack patches, with each matrix corresponding to a different stain. One way to represent this conditioning is:

2 $$\tilde c = \left[ {c_1,c_2,c_3} \right]$$

where [·] denotes concatenation and *c*_*i*_ represents a 256 × 256 matrix of labels for the *i*-th stain type (in this example, H&E, Masson’s trichrome or Jones’ silver). For a pair of input and target images from the *i*-th stain dataset, *c*_*i*_ was set to be an all-one matrix, while all remaining matrices were assigned values of zero.

**Fig. 6:**
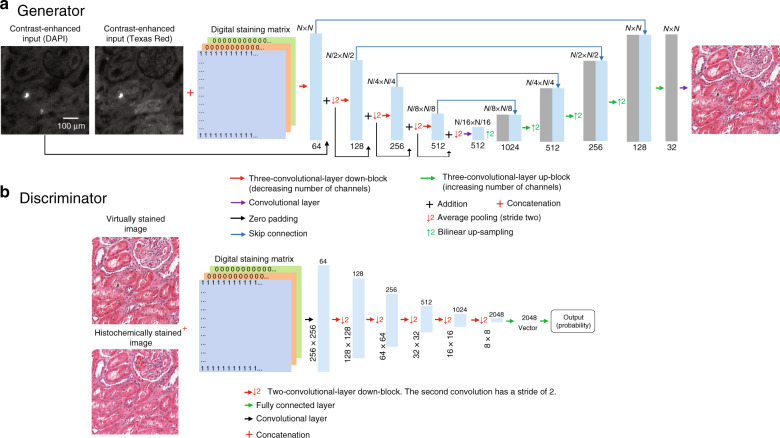
Diagram showing the network architecture of the GAN used to perform the transformation. **a** Generator network. **b** Discriminator network

A GAN is composed of two deep neural networks, a generator and a discriminator (Fig. 6). During GAN training, the generator learns to perform a statistical transformation to generate a virtually stained image, while the discriminator attempts to distinguish between histochemically stained images and their virtually stained counterparts. The networks improve by learning from one another, improving the quality of the virtually stained images. For this task, we defined the loss functions of the generator and discriminator as:

3 $$\begin{array}{l}\ell _{\mathrm{generator}} = L_1\left\{ {{\it{z}}_{\mathrm{label}},G\left( {x_{\mathrm{input}},\tilde c} \right)} \right\} + \lambda \times {\mathrm{TV}}\left\{ {G\left( {{x}_{\mathrm{input}},\tilde c} \right)} \right\} + \alpha \times \left( {1 - D( {G( {x_{\mathrm{input}},\tilde c}),\tilde c})} \right)^2\\ \ell _{\mathrm{discriminator}} = D\left( {G( {x_{\mathrm{input}},\tilde c}),\tilde c} \right)^2 + \left( {1 - D( {z_{\mathrm{label}},\tilde c})} \right)^2\end{array}$$

where the total variation (TV) operator and mean absolute error (*L*_1_-norm) are used to regularize the generator’s output and ensure that it is highly accurate. The TV operator and the *L*_1_-norm are defined as:

4 $${\mathrm{TV}}\left( z \right) = \mathop {\sum }\limits_p \mathop {\sum }\limits_q |z_{p\, + \,1,q} - z_{p,q}| + |z_{p,q + 1} - z_{p,q}|$$

5 $$L_1\left( {z,G} \right) = \frac{1}{{\mathrm{P} \times {\mathrm{Q}}}}\mathop {\sum }\limits_p \mathop {\sum }\limits_q |z_{p,q} - G\left( {x_{\mathrm{input}},\tilde c} \right)_{p,q}|$$

where *D*(·) and *G*(·) refer to the outputs of the discriminator and generator networks, respectively; *z*_label_ denotes the bright-field image of the histochemically stained tissue; and *x*_input_ represents the input to the neural network. P and Q represent the numbers of vertical and horizontal pixels, respectively, of the image patch, and p and q represent the pixel locations. The regularization parameters (*λ* and *α*) were set to 0.02 and 2000, respectively, to accommodate a total variation loss term of approximately 2% of the *L*_1_ loss and a discriminator loss term of 98% of the total generator loss.

For the generator a modified version of the U-net architecture was adopted^24^, as visualized in Fig. 6a. This U-net consists of four “down-blocks” followed by four “up-blocks”. Each of the down-blocks is made up of three convolutional layers and their activation functions, which together double the number of channels. These convolutional layers are followed by an average pooling layer with a stride and kernel size of two, which effectively down-samples the image. The up-blocks first bilinearly resize the tensors, up-sampling them by a factor of two. This is followed by three convolutional layers and their activation functions. These convolutional layers together reduce the number of channels by a factor of four. Between each of the up- and down-blocks of the same level, a skip connection is used. These skip connections concatenate the output of the down-blocks with the up-sampled values, allowing data to be passed at each level. Following these down- and up-blocks, a convolutional layer is used to reduce the number of channels to three, which correspond to the three colour channels in the bright-field image.

The discriminator network, visualized in Fig. 6b, receives six input channels. Three channels (YCbCr colour map) come from either the generator output or the target/label, and three come from the one-hot-encoded digital staining matrix. The discriminator architecture contains a convolutional layer that transforms this input into a 64-channel feature map, which is, in turn, passed through a set of five blocks, each consisting of two convolutional layers and their corresponding activation functions. The second of these convolutional layers doubles the number of channels and has a stride of two. These five blocks are followed by two fully connected layers, which reduce the dimensionality to a single channel, which is acted upon by a sigmoid activation function.

The convolutional filter size throughout the GAN is set to 3 × 3; the outputs of these filters are acted upon by the Leaky ReLU activation function, which is described as:

6 $${\mathrm{LeakyReLU}}\left( x \right) = \left\{ {\begin{array}{*{20}{c}} \!\!\!\!\!{x\;\mathrm{for}\;{\it{x}}\, > \,0} \\ {0.1x\;\mathrm{otherwise}} \end{array}} \right.$$

During training, the learnable parameters were updated using the adaptive moment estimation (Adam) optimizer with learning rates of 1 × 10^−4^ for the generator network and 2 × 10^−6^ for the discriminator network. For each step of discriminator training, ten iterations of training were performed for the generator network. The batch size for training was set to 8.

### Virtual staining of unlabelled tissue images with a single stain

Once the network had been trained, the one-hot-encoded label $\tilde c$ was used to condition the network to generate the desired stained images. In other words, to generate solely the *i*-th stain, the matrix *c*_*i*_ was set to be an all-one matrix, and the remaining matrices were set to be all zeros.

### Stain blending and micro-structured virtual staining of unlabelled tissue images

Following the training process of the neural network model, we can use the conditional matrices in ways different to that in which the model was trained to virtually create new types of stains. The basic encoding rule that should be satisfied can be summarized as follows:

7 $$\mathop {\sum }\limits_{i = 1}^{N_{\mathrm{stains}}} c_i,_j,_k = 1$$

In other words, for a given set of indices *j* and *k*, the sum over the number of stains on which the network was trained (*N*_stains_ = 3 in our example) should be equal to 1. By modifying the class encoding matrices to use a mixture of multiple classes, the various stains can be blended, creating unique stains with features inherited from the various stains learned by the artificial neural network. Examples of such blended stains are illustrated in Fig. 4.

Another possible use of our trained multistain neural network is to partition the tissue field of view into different regions of interest (ROIs) and virtually stain each ROI using a different specific stain or blend of a sub-set of these stains:

8 $$\mathop {\sum }\limits_{i = 1}^{N_{\mathrm{stains}}} c_i,_j,_k = 1\, \quad\mathrm{for}\;{\it{j,k}} \subseteq \mathrm{ROI}$$

where ROI is the defined region of interest in the sample field of view. Multiple non-overlapping ROIs can be defined across a field of view, with different stains applied to different ROIs or micro-structures. These can be either defined by the user or algorithmically generated. As an example, a user can manually define various tissue areas via a graphical user interface and stain them with different stains. This will result in different tissue constituents being stained differently, as illustrated in Figs. 1 and 3. We have implemented this ROI-selective staining (micro-structured staining) functionality using the Python segmentation package Labelme^25^. Using this package, we can generate logical masks in accordance with labelled ROIs, which are then processed to be the $\tilde c_{ROI}$ labels for specific microscopic areas. Other manual, software or hybrid approaches can also be used to implement the selection of certain tissue structures.

### Single-stain network used for SSIM calculations

To generate virtually stained images using a single-stain network, a network with the same architecture but excluding the digital staining matrix was used. A separate network was trained for each of the three stains using the portion of the dataset specific to that stain. This single-stain network was implemented followed the approach previously reported^15^.

### Implementation details

The virtual staining network was implemented using Python version 3.6.0, with the TensorFlow framework version 1.11.0. We implemented the software on a desktop computer with an Intel Xeon W-2195 CPU @2.30 GHz and 256 GB of RAM running the Microsoft Windows 10 operating system. Network training and testing were performed using a single NVIDIA GeForce RTX 2080 Ti GPU. The network was trained for 21,000 discriminator training steps over 47 h. Using a single GPU, inference can be performed at a rate of 3.9 s per 1 mm^2^ of unlabelled tissue.
